# The Neurobiology of Cannabis Use Disorders: A Call for Evidence

**DOI:** 10.3389/fnbeh.2016.00086

**Published:** 2016-05-10

**Authors:** Valentina Lorenzetti, Janna Cousijn, Nadia Solowij, Hugh Garavan, Chao Suo, Murat Yücel, Antonio Verdejo-García

**Affiliations:** ^1^School of Psychological Sciences and Monash Institute of Cognitive and Clinical Neurosciences, Monash UniversityMelbourne, VIC, Australia; ^2^Addiction Development and Psychopathology Lab, Department of Psychology, University of AmsterdamAmsterdam, Netherlands; ^3^School of Psychology, Centre for Health Initiatives and Illawarra Health and Medical Research Institute, University of WollongongWollongong, NSW, Australia; ^4^Department of Psychological Science, College of Arts and Sciences, The University of VermontBurlington, VT, USA

**Keywords:** neurobiology, cannabis use disorder, dependence, addiction, stress, physiological

## Cannabis use disorders, why bother?

Using cannabis is perceived by many as relatively harmless, but the adverse effects of problematic cannabis use are significant. Thirteen million individuals globally have Cannabis Use Disorders (CUDs; UNODC, 2015), with relapse rates comparable to those of other substance use disorders (~52–70%; Budney et al., 1999; Chauchard et al., 2013). Contrasting non-problematic recreational cannabis use, severe forms of CUD involve compulsive use despite significant harms to mental health; high stress levels (craving, withdrawal); cognitive deficits; academic and work absenteeism; and significant risky behaviors, such as driving and operating machinery while intoxicated. Worryingly, the concentration of Δ^9^-tetrahydrocannabinol, the compound driving the addiction liability of cannabis, has risen in cannabis products over the past decade (UNODC, 2015).

## Neurocognitive mechanisms underlying CUDs: what we know and what we do not know

Mounting (although mixed) evidence shows that regular cannabis use is linked to abnormal neurobiology (Lorenzetti et al., 2014) in regions subserving reward, craving/urges, and cognitive control—key components of addiction (Everitt and Robbins, 2005). Neurobiological studies of CUDs specifically are sparse and limited because diagnostic assessments of CUDs are rarely performed (Lorenzetti and Cousijn, 2015). There is a strong need to study CUDs, given their high prevalence and treatment demands, and worldwide trends toward legalizing cannabis products.

Emerging evidence shows neuroadaptations in cannabis users with CUDs (Lorenzetti and Cousijn, 2015), which may be related to the development of addictive behaviors (i.e., reduced prefrontal control, alteration in reward systems, and increased stress, anxiety, and withdrawal). CUDs may exacerbate and expand the neurobiological alterations associated with pre-existing general vulnerability to drug use and recreational cannabis use, particularly in addiction-relevant areas (Ersche et al., 2010). Neurobiological models propose mechanisms of addiction related neuroadaptations within distinct brain regions in the transition from recreational, non-problem, and reward-driven drug use (ventral striatum, medial prefrontal cortex) to compulsive and habitual drug use (dorsal striatum, lateral prefrontal cortex, stress circuit; Everitt and Robbins, 2005, 2013). Specific aspects of severe CUDs (e.g., neural signatures of withdrawal, stress, craving, and compulsive use) may dissociate from the direct effects of cannabis exposure in milder forms of CUDs (e.g., possibly limited to brain areas high in cannabinoid receptors). This notion is yet to be tested. Neurobiological models of addiction and the transition from regular heavy use to compulsive drug use mostly rely on preclinical evidence from artificially induced drug taking of substances other than cannabis such as cocaine (Ito et al., 2002; Vanderschuren et al., 2005; See et al., 2007). Emerging evidence in human cocaine users has elucidated neural network changes involved in compulsive drug use at the level of frontal-striatal circuits and these neural changes have been linked to drug relapse, validating preclinical models of drug addiction (Contreras-Rodríguez et al., 2015; Hu et al., 2015). Supportive evidence for these models also comes from a study of alcohol dependent individuals (Vollstädt−Klein et al., 2010) and functional imaging studies in cannabis users (Filbey and Dunlop, 2014; Vingerhoets et al., 2016) implicating frontal–striatal pathways. Most human studies of cannabis users, however, compare groups of heavy cannabis users with varying levels of cannabis related problems to controls without assessing CUD severity with rigorous diagnostic instruments. As such, little is known about the neurobiology underlying cannabis addiction.

Addiction-specific neural alterations—rather than those associated with use *per se*—are likely to predict negative outcomes in cannabis users. Consistent with this notion, dependent users have worse mental health outcomes than non-dependent users (Van der Pol et al., 2013b,c). Moreover, severity of cannabis use-related problems—rather than quantity of use—predicts activity in reward-related brain regions in response to cannabis cues, which is a well-validated measure of craving (Cousijn et al., 2013). Uncovering whether CUDs involve neuroadaptations dissociable from those linked to recreational, non-problem cannabis use is critical.

## Suggestions for future studies

Important steps to resolve addiction vs. exposure dependent alterations in cannabis users include the characterization of the neural, behavioral, polygenic risk markers, and epigenetics (Sherva et al., 2016; Walters and Owen, 2016) that identify (i) which recreational cannabis users or cannabis naïve individuals are vulnerable to develop a CUD; (ii) the recreational non-problem cannabis users en route to develop a severe CUD; (iii) which users have persistent severe CUDs (Van der Pol et al., 2013a); (iv) which users transit to a lower severity CUD/non-problem cannabis use/abstinence; and (v) sex differences in the development of CUDs, as females are underrepresented in the existing literature, while presenting a distinct profile from males (e.g., faster transition to dependence, more treatment resistant; Lorenzetti and Carter, 2015).

Twin studies are required to address the above research question (i) and disentangle epigenetic factors that confer risk to develop CUDs in some but not others (Gillespie et al., 2009). Large-scale longitudinal studies are warranted to address research questions (i)–(v) and track young adolescents yet naïve to cannabis through to late adulthood. Such studies should combine diagnostic cutoffs that inform on the clinical significance of cannabis use (i.e., DSM 5, ICD-10); self-reported detailed information on cannabis use (daily/almost daily use, duration, and age of onset); objective (i.e., biological specimens) quantification of cannabinoids (Lorenzetti et al., 2016); cognitive and brain anatomy/function (using MRI); and recruitment approaches that maximize sample representativeness (e.g., the general community, coffee shops; Van der Pol et al., 2011). Notable examples include the Netherlands XTC Toxicity (NeXT) study (De Win et al., 2005), the USA ABCD study http://addictionresearch.nih.gov/abcd-study and the Dutch Cannabis Dependence Study (CanDep; Van der Pol et al., 2011).

As longitudinal studies are very expensive and time consuming, we can address relevant research questions (i)–(vi) in a timely fashion, via re-examination, online data sharing, and follow up from already collected neuroscientific datasets on cannabis using cohorts with varying levels of problems with use. Problem vs. recreational users may be segregated (to enable their comparison) using information available from each study's instruments, on key addiction phenotypes (e.g., craving, withdrawal, difficulties in controlling use, persistent use despite harmful consequences on mental health, and socio-occupational functioning, higher priority given to drug use than to other activities and obligations; APA., 2013; WHO, 2016). A notable example of this includes the internationally coordinated initiative ENIGMA Addiction Working Group (Mackey et al., 2016).

Finally, we propose the development of an agreed-upon instrument to objectively assess key features of cannabis addiction vs. non-problem use in neuroscientific settings, as most measures of cannabis related problems are borrowed from those used for other substances, the features of which may not fit those relevant to cannabis dependence (Lorenzetti et al., 2016). To this end, a Delphi review (Dalkey and Helmer, 1963) with world-class experts in the clinical/neuroscientific aspects of cannabis and substance dependence may be conducted and may identify additional hot topics in CUDs.

In conclusion, we call for a greater, systematic and coordinated research effort internationally, to identify pathways in and out of CUDs and fill the existing gap between the limited knowledge on CUDs and the increasing availability of cannabis products.

## Author contributions

VL and JC formulated the key concepts and led the writing of the commentary; NS, HG, CS, and MY contributed to the concepts and writing of the commentary. AV contributed to the key concepts and to the writing of the commentary.

## Funding

NS is supported by the Australian Research Council Future Fellowship FT110100752. MY is supported by the National Health and Medical Research Council of Australia, Senior Research Fellowship.

### Conflict of interest statement

The authors declare that the research was conducted in the absence of any commercial or financial relationships that could be construed as a potential conflict of interest.
